# Imaging Evaluation of Thymoma and Thymic Carcinoma

**DOI:** 10.3389/fonc.2021.810419

**Published:** 2022-01-03

**Authors:** Chad D. Strange, Jitesh Ahuja, Girish S. Shroff, Mylene T. Truong, Edith M. Marom

**Affiliations:** ^1^ Department of Thoracic Imaging, University of Texas MD Anderson Cancer Center, Houston, TX, United States; ^2^ Department of Diagnostic Radiology, Chaim Sheba Medical Center, Tel Aviv University, Tel Aviv, Israel

**Keywords:** thymoma, thymic carcinoma, CT, MRI, PET/CT

## Abstract

Imaging is integral in the management of patients with thymoma and thymic carcinoma. At initial diagnosis and staging, imaging provides the clinical extent of local invasion as well as distant metastases to stratify patients for therapy and to determine prognosis. Following various modalities of therapy, imaging serves to assess treatment response and detect recurrent disease. While imaging findings overlap, a variety of CT, MRI, and PET/CT characteristics can help differentiate thymoma and thymic carcinoma, with new CT and MRI techniques currently under evaluation showing potential.

## Introduction

Imaging plays several roles in the management of patients with thymoma and thymic carcinoma. Imaging is integral in the initial diagnosis and staging of patients, emphasizing the detection of locally invasive disease and distant metastases, to properly stratify patients for therapy, and to establish prognosis. Following various modalities of treatment, imaging serves to assess therapy response and to identify recurrent disease. This is particularly important in that patients with resected recurrent disease have similar outcomes as patients without recurrence (1).

## Routine Imaging Modalities

**Figure 1:**
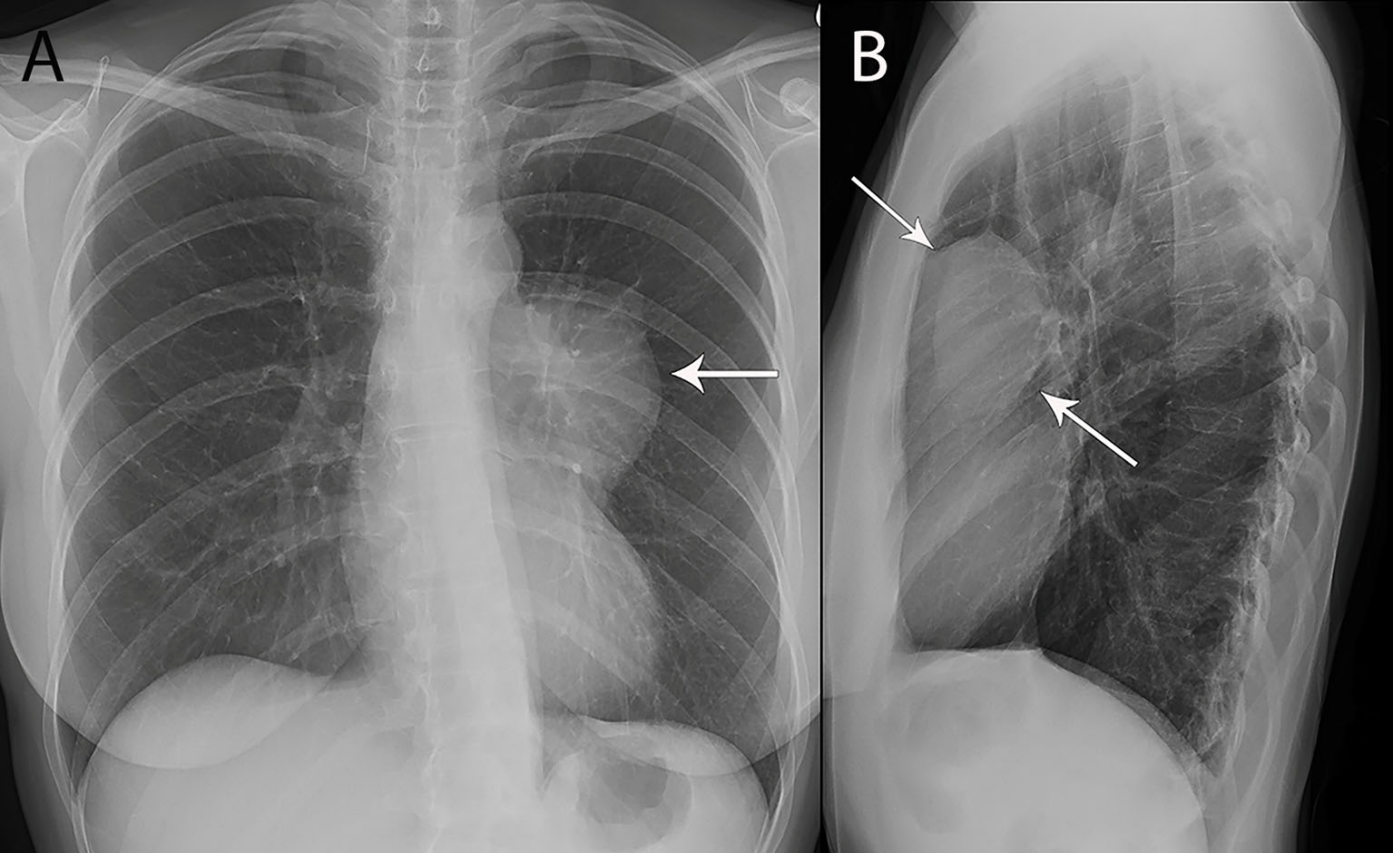
53 year old woman with thymoma. **(A, B)** Frontal chest radiograph **(A)** shows left mediastinal contour abnormality (arrow) that results in loss of the silhouette of the upper left heart border. **(B)** The lesion is localized to the prevascular mediastinum in the retrosternal space (arrows) on the lateral chest radiograph **(B)**.

Chest radiographs are the most commonly performed imaging examination and can be the first modality to suggest a thymic mass. Larger thymic tumors can result in extra soft tissue projecting over normal anatomic structures. Radiographically, thickening of the anterior junction line can signal a thymic tumor in the prevascular space. Additionally, the “silhouette sign” is another useful radiographic sign. In patients with a normal chest radiograph, the air in the lungs delineates the structures that abut the lung, such as the heart or the mediastinum. When a mass is present, clear delineation of anatomic structures is limited given that the mass now abuts the normal structure instead of air. As the mass and normal mediastinal structures are of similar densities, thus cannot be distinguished one from another, this results in the obscuration of structures or loss of their silhouette, designated the “silhouette sign.” The lateral radiograph can help confirm the presence of thymic tumors in the prevascular space. The prevascular space is readily seen on the lateral radiograph behind the sternum and is normally lucent. When a mass is present in this space, it is dense and has a sharp border if the lung abuts it (**Figure 1**). It should be noted, however, that smaller prevascular lesions are often not radiographically apparent. Low sensitivity and specificity of routine radiographs in prevascular tumor detection offsets the advantages of low cost and low radiation exposure; therefore, cross-sectional imaging is invariably utilized.

**Figure 2:**
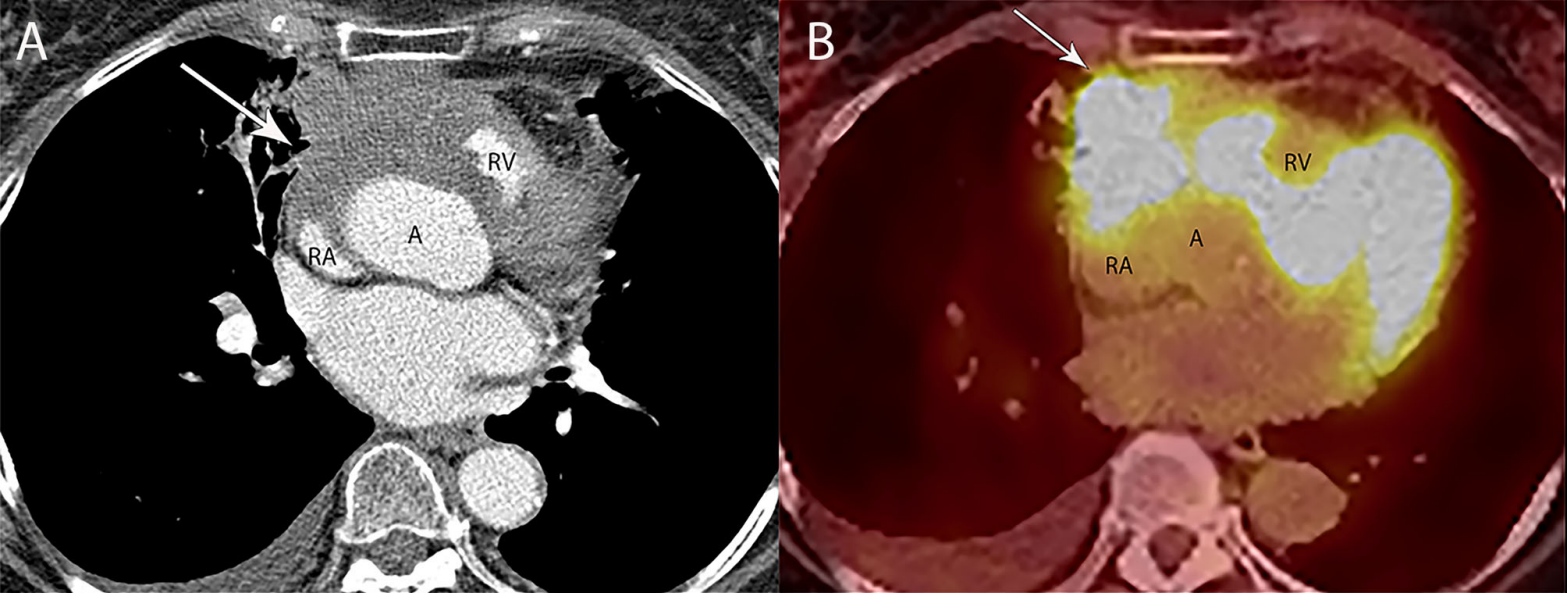
80 year old woman with adenosquamous thymic carcinoma. **(A)** Contrast-enhanced CT shows right prevascular mediastinal tumor (arrow) invading the pericardium between the right atrium (RA), ascending aorta **(A)** and right ventricle (RV). **(B)** PET/CT shows the tumor is markedly FDG avid.

**Figure 3:**
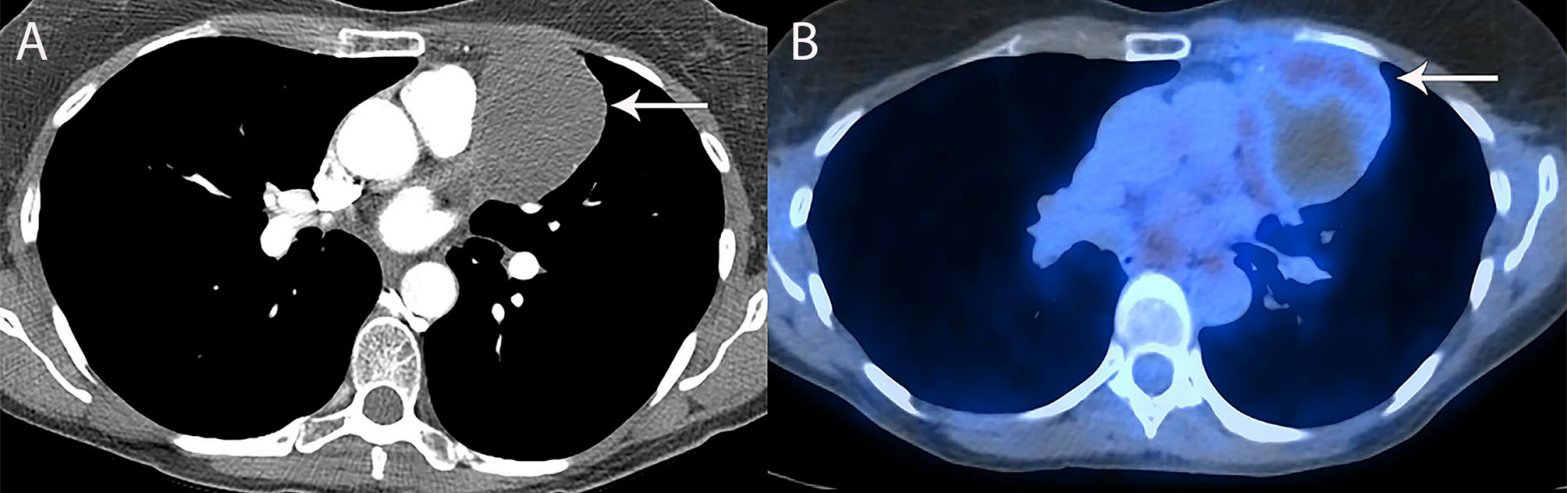
53 year old woman with thymoma. **(A)** CT shows the left prevascular mediastinal mass (arrow) is mostly homogenous, with low attenuation measuring 10 Hounsfield Units suggesting a cystic component, yet has a denser component of 48 Hounsfield Units along the anterior peripheral aspect suggesting some solid component. **(B)** PET/CT shows FDG uptake with SUVmax of 3.4 along the anterior peripheral aspect of the mass (arrow). At resection, pathology showed thymoma with cystic component and no invasion of the pericardium or lung.

Contrast enhanced computed tomography (CT) is the imaging modality of choice for imaging thymic tumors due to its high spatial and temporal resolution, ease of access, and convenience (2). CT can discern location, morphology, shape, margins, size, density, enhancement, and relationship to, or invasion of, adjacent structures (3) (**Figure 2**). Overall, CT has been found to be equal or superior to magnetic resonance imaging (MRI) in the evaluation of mediastinal masses with the exception of cysts or cystic components of tumors (4) (**Figure 3**).

**Figure 4:**
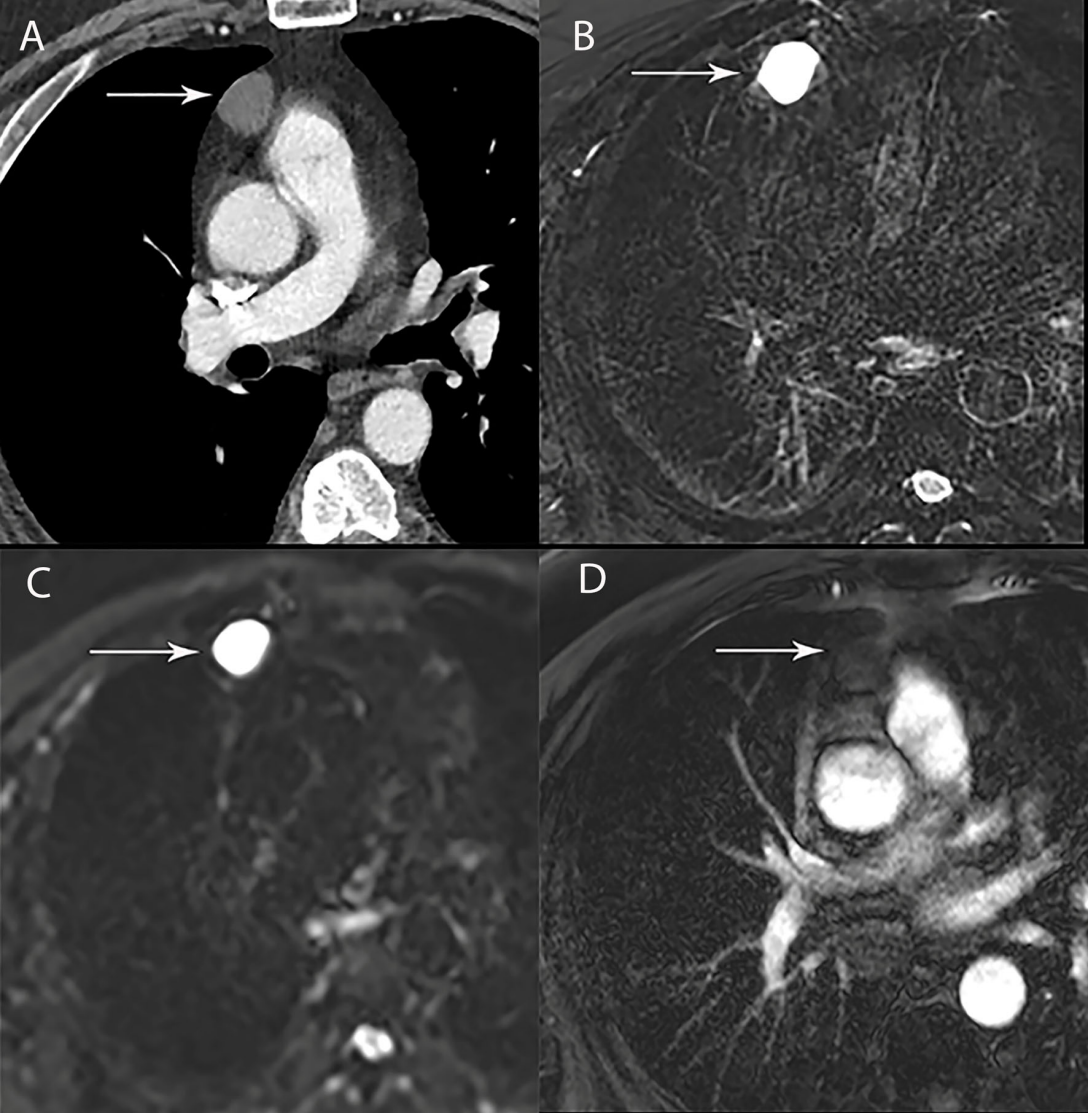
67 year old man with right thymic cyst. **(A)** Contrast-enhanced CT shows a right prevascular mediastinal 2.2 cm lesion (arrow) with 34 Hounsfield units, which can represent solid or cystic lesion with proteinaceous material or hemorrhage. **(B–D)** MRI is useful to determine that this is a simple thymic cyst (arrow) with high signal intensity on T2 weighted **(B)** and short tau inversion recovery (STIR) **(C)** and no enhancement on the post contrast images **(D)**.

**Figure 5:**
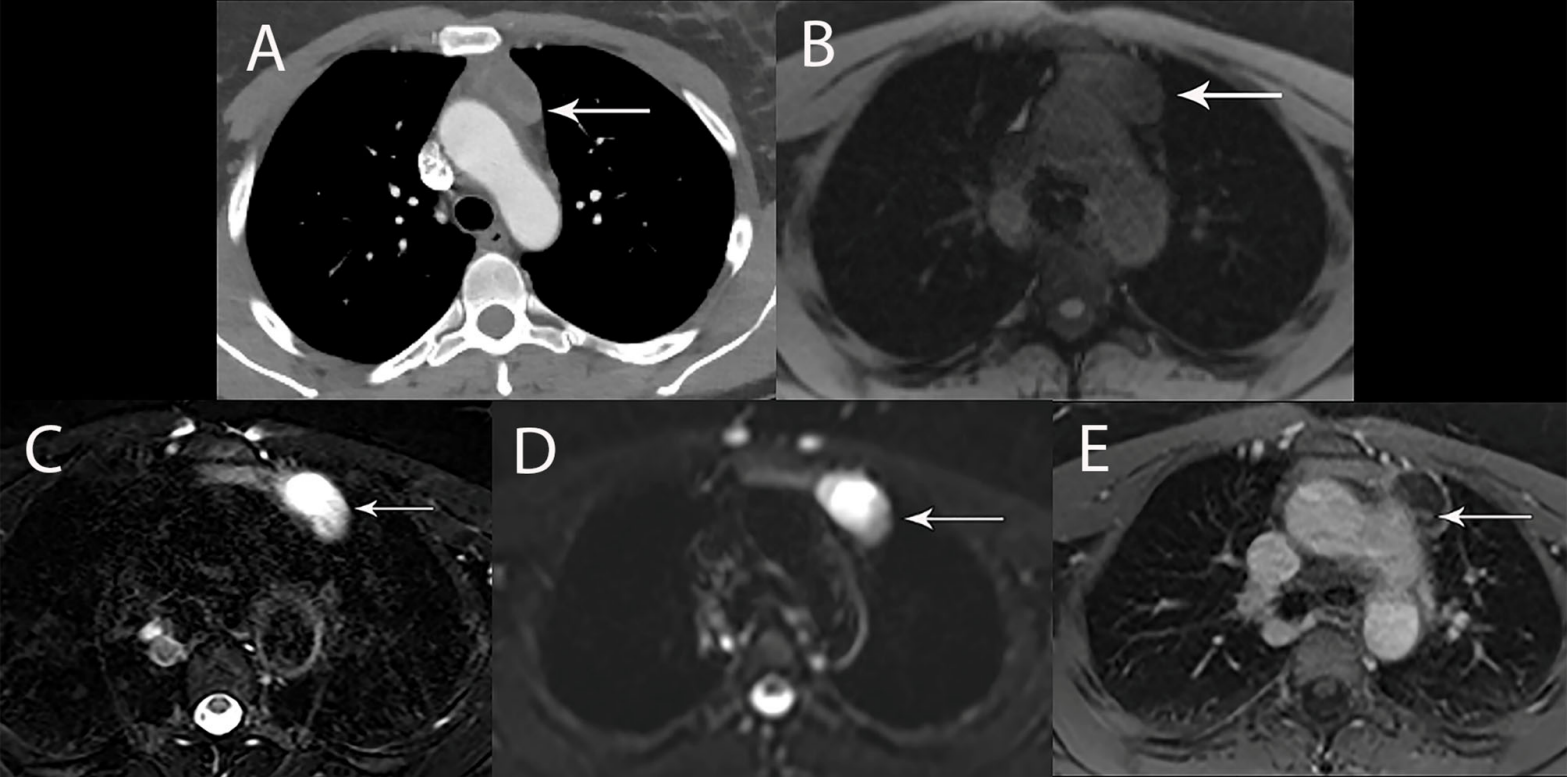
44 year old woman with cystic thymoma. **(A)** Contrast enhanced CT shows solid component along the posterior aspect. MRI showed intermediate signal intensity on T1 weighted **(B)**, high signal intensity on T2 weighted images **(C)** and DWI **(D)** consistent with cystic component. The small solid component (arrow) shows enhancement on post contrast T1 weighted images **(E)**.

While MRI is not routinely used in the evaluation of thymic tumors, there are certain scenarios where it is uniquely helpful, such as to distinguish solid from cystic lesions when CT is equivocal, to evaluate cystic or necrotic components of a mass, to evaluate for enhancing septae within cystic lesions, and to evaluate for areas of subtle local invasion (**Figures 4**, **5**). Additionally, chemical shift imaging can be utilized to detect microscopic or intravoxel fat, which can help differentiate thymic neoplasm from benign thymic hyperplasia (5–7). MRI can be performed when limiting radiation exposure is of concern, such as in younger patients. Finally, unenhanced MRI can be performed in patients who cannot receive iodinated CT contrast due to contrast allergy or poor renal function.

Fluorodeoxyglucose (FDG) positron emission tomography (PET)/computed tomography (CT) has a complicated and incompletely defined role in the evaluation of thymic masses. Some of the confounding variables related to FDG PET/CT usage include false-positive results, meaning FDG uptake within a non-neoplastic mass, which can be seen in infection, thymic hyperplasia, fibrosing mediastinitis, and other non-neoplastic processes. On the other hand, lack of increased FDG uptake is seen in some histological types of thymic malignancy, and there is lack of standardization in techniques which can result in quantitative variability between studies (8). Other neoplasms found in the prevascular mediastinum are also FDG avid, such as lymphoma or malignant germ cell tumors. Thus the presence of FDG uptake in a prevascular mass cannot distinguish between a thymic epithelial tumor and other tumors which commonly occupy this anatomical area. FDG uptake has been studied to predict tumor invasiveness and prognosis. Some studies reported FDG PET/CT as useful in differentiating low-grade from high-grade thymic malignancies, while other studies reported these observations as controversial owing to overlap in imaging findings and FDG uptake between low-grade and high-grade thymic tumors (9). The role of PET/CT is more clear in aggressive tumors, such as thymic carcinoma, due to higher overall tumor metabolism, with studies indicating that a maximum standard uptake value (SUVmax) of 6 possibly serving as a cutoff to separate thymic carcinoma from lower grade thymic tumors (10) (**Figures 2**, **3**). Finally, PET/CT has a role in the detection of occult metastasis when the tumor is FDG avid.

## Staging

While fifteen different staging systems have been proposed over the years, thymic tumors have predominately been staged using the Masaoka-Koga staging system as it has been shown to correlate with survival (11–13). Masaoka-Koga staging is based on gross and microscopic tumor properties with Stage 1 tumors being completely encapsulated; Stage II tumors demonstrating microscopic invasion through the capsule (IIa) or macroscopic invasion into surrounding fat (IIb); Stage III tumors invading into adjacent structures such as lung, great vessels, or pericardium; and Stage IV tumors demonstrating pleural or pericardial dissemination (IVa) or lymphatic-hematogenous metastasis (IVb) (1). Issues with this staging scheme were that it relied on a small series, of 96 patients from one institution, and was difficult to implement. A capsule was not always present or complete making implementation of the stage II problematic at pathology and impossible to see with imaging at clinical staging.

Given the need to more accurately stage patients prior to treatment (clinical staging) and the need to find a staging system with greater consistency at pathologic examination, as well as proven as a prognostic determinant on a large patient population from multiple institutions, the International Association for the Study of Lung Cancer (IASLC), the International Thymic Malignancies Interest Group (ITMIG), the European Society of Thoracic Surgeons, the Chinese Alliance for Research on Thymomas, and the Japanese Association of Research on Thymus partnered together to develop a TNM staging system for thymic tumors. While the Masaoka-Koga staging system was derived from retrospective series of only 96 patients, the retrospective database of thymic tumors included nearly 10,000 cases (14, 15). These cases were collected for the eighth edition of TNM classification for malignant tumors which has now been adopted by the American Joint Committee on Cancer and the Union for International Cancer Control (16).

In the new TNM classification system for thymic tumors, the T descriptor describes local invasion, and not size of tumor, as size was not found to be a prognostic factor, with T1 tumors demonstrating invasion into the mediastinal fat (T1a) or mediastinal pleura (T1b), T2 tumors demonstrating invasion into the pericardium, T3 tumors demonstrating invasion into the lung, brachiocephalic vein, superior vena cava, chest wall, or phrenic nerve, and T4 tumors demonstrating invasion into the aorta, intrapericardial pulmonary artery, myocardium, trachea, or esophagus. The N descriptor distinguishes anterior/perithymic lymph nodes as N1 and deep intrathoracic or cervical lymph nodes as N2. Pleural and pericardial nodules represent M1a disease and distant organ metastasis represent M1b disease (17–19). While the ultimate assigned stage may be the same with the TNM and Masaoka-Koga staging systems, TNM allows for a more detailed breakdown and reporting of the extent of disease (16). A few highlighted differences help contrast the two staging systems. In TNM, capsular and mediastinal pleural invasion are T1 disease, and in the absence of nodal disease, stage I, compared with stage II disease in Masaoka-Koga. Pericardial invasion is TNM T2/stage II instead of Masaoka-Koga stage III. Anterior/perithymic nodal involvement has been downgraded from Masaoka-Koga stage IVb to TNM stage IVa.

Ried et al. studied 76 patients to compare the Masaoka-Koga staging system with the recently proposed TNM staging system. They found that a number of Masaoka-Koga stage IIa and IIb tumors were reclassified as TNM stage I. Additionally, they reported the TNM system more accurately characterized the more heterogeneous Masaoka-Koga stage III disease to determine which patients were better surgical candidates. They concluded that the TNM staging system was overall clinically useful and applicable as compared with the Masaoka-Koga staging system (20).

A few limitations of the new TNM staging system for thymic tumors have been discussed, however. The number of patients who had nonsurgical treatment was small which could hinder staging predictive ability with higher stage disease. The nodal map was derived from Japanese practice patterns which are more empirically based with more consideration on feasibility of surgical sampling. Finally, due to the limited data available, differentiation between parenchymal nodules (M1b) and pleural/pericardial nodules (M1a) was made empirically, not statistically (16).

## Assessment of Treatment Response

The most commonly utilized method to assess response to treatment is the Response Evaluation Criteria in Solid Tumors (RECIST) version 1.1. ITMIG does, however, recommend certain modifications and caveats to the use of RECIST version 1.1 in thymic tumors due to unique patterns of spread (8). First, ITMIG recommends pretreatment and posttreatment image interpretation be done by the same experienced radiologist to decrease interobserver variability when assessing the often large irregular tumors, vague borders, and local infiltration (21, 22). Second, because thymic epithelial tumors tend to spread along the pleura, ITMIG recommends using a modification of RECIST similar to that used for malignant pleural mesothelioma (MPM) (8, 12).

A summary of RECIST version 1.1, using the modification for MPM, would involve measuring up to a maximum of two lesions per organ and five lesions in total, representing all involved organs, as target lesions at baseline. Longest diameter measurements are used, with the exception of pleura and lymph nodes which are measured in short axis. When the pleura is used as a target organ, short axis (or perpendicular to the pleura) measurements are taken at two locations at three separate CT levels separated by at least 1 cm. The sum of these pleural measurements, up to a maximum of 6, is the overall pleural measurement, which is then added to non-pleural target lesions up to a total of five (8, 23).

The National Comprehensive Cancer Network (NCCN) recommends a surveillance regimen of CT of the thorax every six months for two years, then annually for ten years in thymoma and annually for five years in thymic carcinoma. Alternatively, ITMIG recommends surveillance frequency for patients after resection of any thymic tumor as annual CT of the thorax for five years following resection. From years six to eleven alternating yearly CT and chest radiograph is performed, and then yearly chest radiographs thereafter. For resected stage III or IVa thymoma, thymic carcinoma, incomplete resection, or other high-risk tumors, additional CT of the thorax is recommended every six months for the first three years with consideration of CT one to three months after surgery to obtain a new baseline after post-surgical inflammation has resolved (12, 24).

CT is the recommended modality of choice for tumor reassessment given that it is the most reproducible (25). In younger patients and in those who cannot be given CT contrast due to allergy or renal function, MRI can be utilized for tumor reassessment. Kerpel et al. evaluated 22 of 187 patients who underwent resection for thymic epithelial tumors to assess the accuracy of MRI compared with CT for follow-up assessment. They concluded that MRI was an adequate alternative to CT for reassessment with the caveat that in patients with sternotomy wires alternating CT with MRI was recommended given associated artifact (24).

In patients with R0 resection (microscopically margin negative resection) or in patients who demonstrate complete radiologic response to therapy, local recurrence in the prevascular mediastinum is defined as tumor in the thymic bed, pericardial, pleural, or pulmonary parenchymal tumor that is immediately adjacent to the thymic bed, lymph nodes immediately adjacent to thymic bed, or in the site of previous noncontiguous metastasis. Regional recurrence is defined as intrathoracic recurrence that is not contiguous with the thymic bed such as parietal pleural nodules, pericardial nodules, visceral pleural nodules, and lymph nodes not contiguous with the thymic bed. Distal recurrence is defined as extrathoracic recurrence or intraprenchymal pulmonary nodules that are not contiguous with the thymic bed (12).

## Routine Imaging Characteristics of Thymic Tumors

The thymus normally appears as a triangular shaped structure in the prevascular space; however, a variety of normal morphological shapes may be seen. Benign and malignant pathologic processes can alter the size and shape of the thymus with considerable overlap which can pose a diagnostic dilemma. Generally, a variety of benign processes can be easily distinguished based on imaging characteristics. A brief review of benign entities will serve as a comparison to the more difficult to distinguish malignant thymic tumors.

## Benign

### Thymic Cysts

Congenital thymic cysts arise from remnants of the thymopharyngeal duct which can occur anywhere along the course of the thymic descent, but most often occur in the prevascular mediastinal space (26). Acquired thymic cysts are more common and are often multi-locular, complex, associated with neoplasm (such as thymoma, lymphoma, or germ cell tumors), radiation therapy, Sjogren syndrome, aplastic anemia, systemic lupus erythematosus, myasthenia gravis, acquired immune deficiency syndrome (AIDS) in children, and can occur after tumor resection (26, 27). In general, on CT, thymic cysts present as well circumscribed round or oval lesions in the prevascular space with fluid density Hounsfield units (HU) under 20, with no thickened, irregular, or enhancing walls. If HU are indeterminate, or if there is a question of enhancement, MRI can be helpful for further evaluation. On MRI, thymic cysts demonstrate increased T2 signal, variable T1 signal depending on protein content, and no appreciable wall or nodular enhancement (**Figure 4**).

### Thymic Hyperplasia

**Figure 6:**
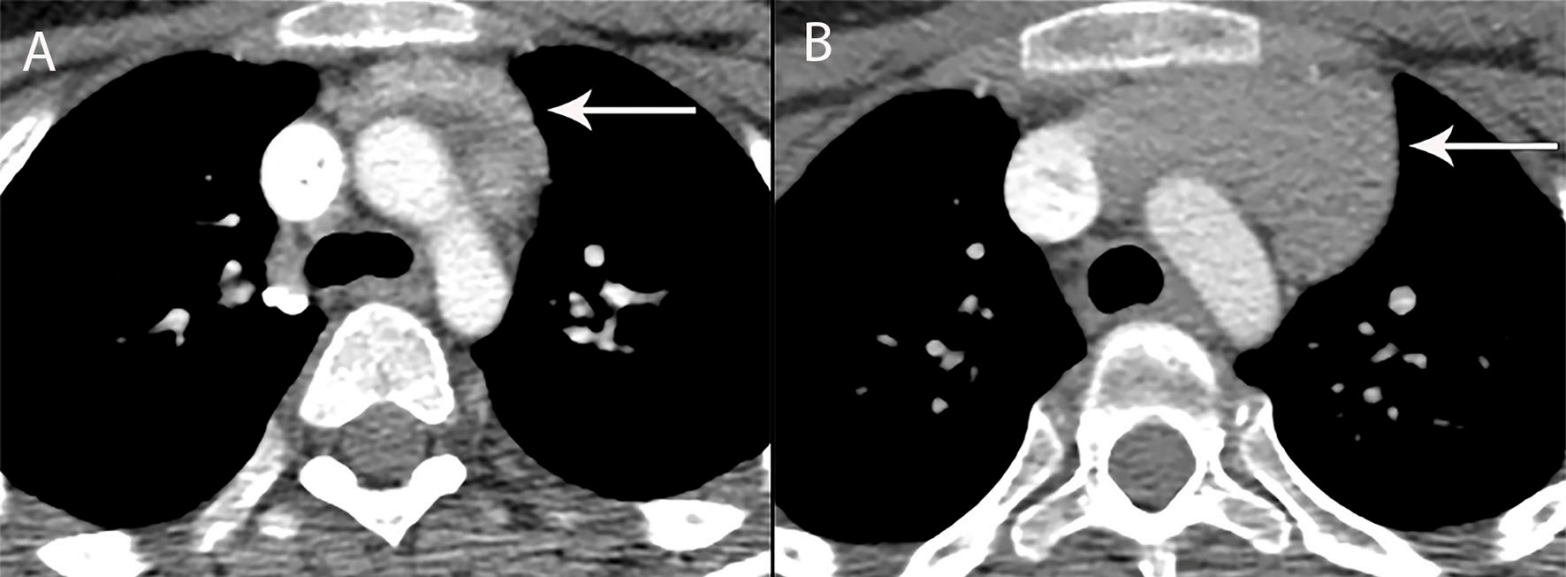
13 year old boy with chondroblastic osteosarcoma of the femur, treated with methotrexate, doxorubicin, cisplatin **(A)** CT shows the normal thymus (arrow) at baseline. **(B)** CT 4 months later shows increase in size of the thymus consistent with rebound hyperplasia (arrow). Enlargement of the thymus gland due to hyperplasia during the recovery phase from physical stress such as after chemotherapy or recovering from burns, does not displace or change the contour of vessels surrounding it. In the appropriate clinical context of thymic hyperplasia, CT is adequate for diagnosis and MRI is not needed for confirmation.

**Figure 7:**
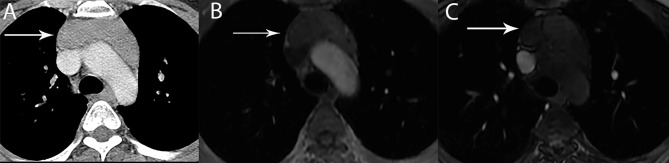
42 year old woman with Grave’s disease and lymphoid thymic hyperplasia. **(A)** Contrast-enhanced CT shows mass-like thymic enlargement (arrow). With such an appearance, thymic hyperplasia, a thymic epithelial neoplasm or lymphoma involvement of the thymus cannot be distinguished one from another. **(B, C)** MRI with in and out of phase imaging shows drop in signal intensity consistent with thymic hyperplasia (arrow), obviating the need for further investigation or biopsy.

True thymic hyperplasia, often referred to as “rebound” hyperplasia, is present when the thymic volume is increased by more than 50%, and is commonly seen after infection, surgery, burns, chemotherapy, radiation therapy, or steroid therapy (**Figure 6**). Lymphoid/follicular hyperplasia is present when there is an increase in the number of lymphoid follicles which is commonly associated with autoimmune diseases, myasthenia gravis (**Figure 7**), and human immunodeficiency virus infection (27–30). In thymic hyperplasia, there is generally symmetric thymic enlargement with smooth contour and margins; however, nodular or bulky appearance can be seen which cannot be readily distinguished from malignancy. In equivocal cases, MRI utilizing in-phase and out-of-phase gradient-echo sequences is performed to identify thymic hyperplasia. Loss of thymic signal during out-of-phase imaging corresponds with microscopic or intravoxel fat, which confirms thymic hyperplasia instead of thymic mass (31) (**Figure 7**).

### Thymolipoma

**Figure 8:**
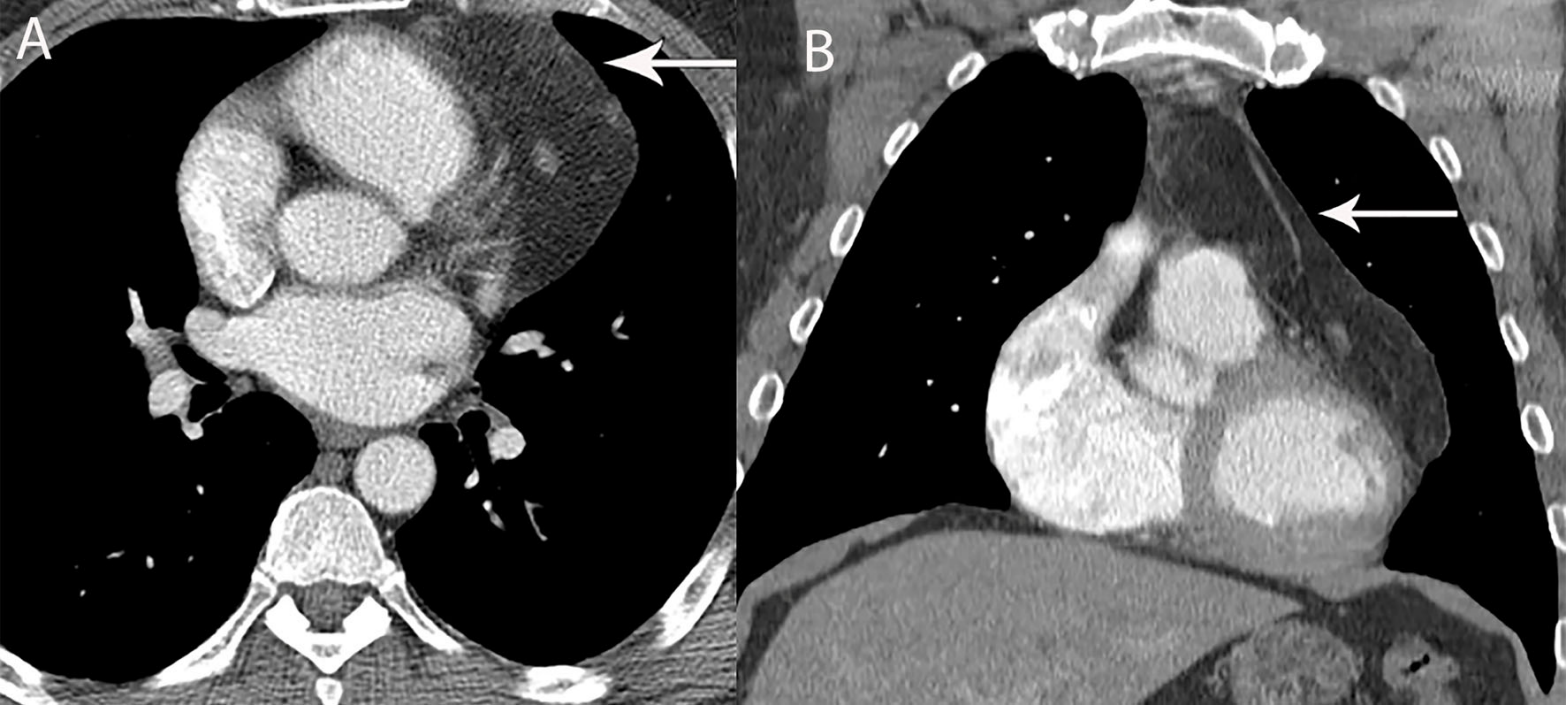
49 year old man with thymolipoma. **(A, B)** Axial and coronal contrast-enhanced CT shows large left prevascular mediastinal lesion with fat attenuation (arrow).

Thymolipoma is a benign, often large, slow growing tumor that arises from the thymus gland. Thymolipomas are composed mainly of adipose tissue with scattered soft tissue/thymic tissue interposed (32, 33). The classic CT appearance of a thymolipoma is a very large predominantly fat density mass in a cardiophrenic angle (**Figure 8**). Occasional symptoms related to compression or displacement of adjacent structures and association with Graves’ disease, myasthenia gravis, and hematological disorders may be seen (3, 30).

### Malignant

#### Thymic Epithelial Tumors

Thymic epithelial neoplasms, including thymoma, thymic neuroendocrine tumor/carcinoid, and thymic carcinoma are predominantly prevascular mediastinal masses which can have a myriad of imaging findings, including homogeneous or heterogeneous, solid or solid/cystic, and well-circumscribed or irregular borders. While there is significant imaging overlap between various grades of thymoma and between thymoma and thymic carcinoma, there are clinical and imaging patterns that emerge that aid in differentiation. Malignant thymic tumors have a median age at presentation in the six decade, while more benign processes have a median age in the fourth to fifth decades (34–36). Clinical symptoms such as pain or shortness of breath are more common in higher grade and malignant tumors (36). Finally, benign tumors more often demonstrate intralesional fat, are midline, and retain normal thymic triangular shape, while malignant tumors are often larger and are more likely to be locally invasive (37–39). Imaging generalizations will be reviewed, followed by techniques being utilized and studied to more accurately differentiate various thymic tumors.

### Thymoma

**Figure 9:**
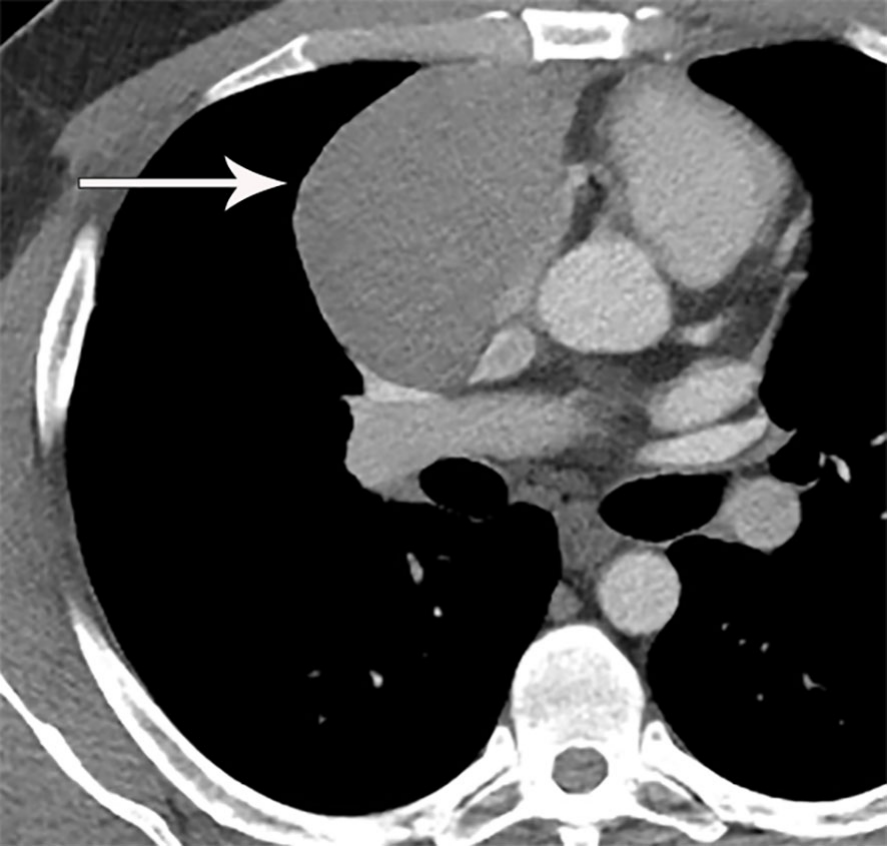
32 year old man with thymoma and myasthenia gravis. Contrast-enhanced CT shows right prevascular mediastinal mass (arrow).

**Figure 10:**
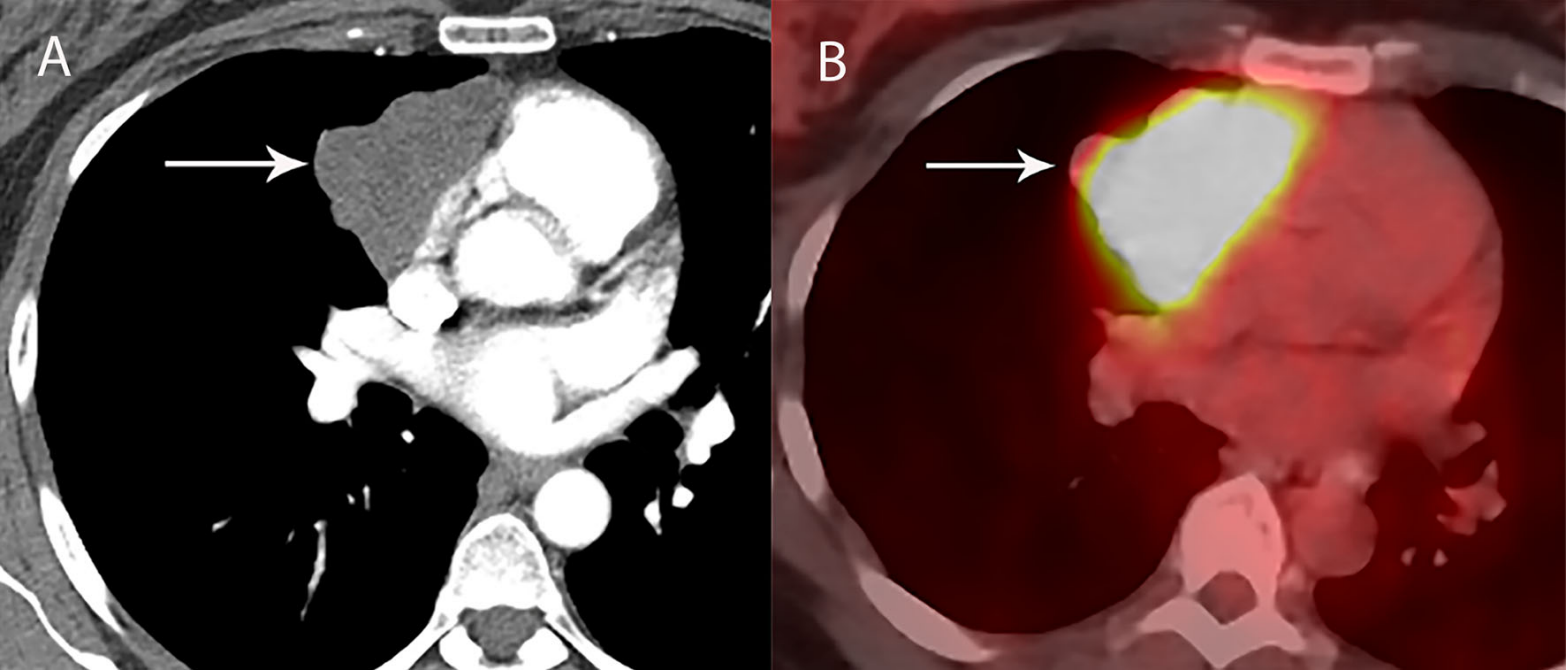
43 year old woman with thymoma. **(A)** Contrast-enhanced CT shows right prevascular mediastinal mass (arrow). **(B)** FDG PET/CT shows FDG avid thymoma (arrow) with SUV of 16. Presence of intense FDG uptake suggests more aggressive type thymoma or thymic carcinoma.

**Figure 11:**
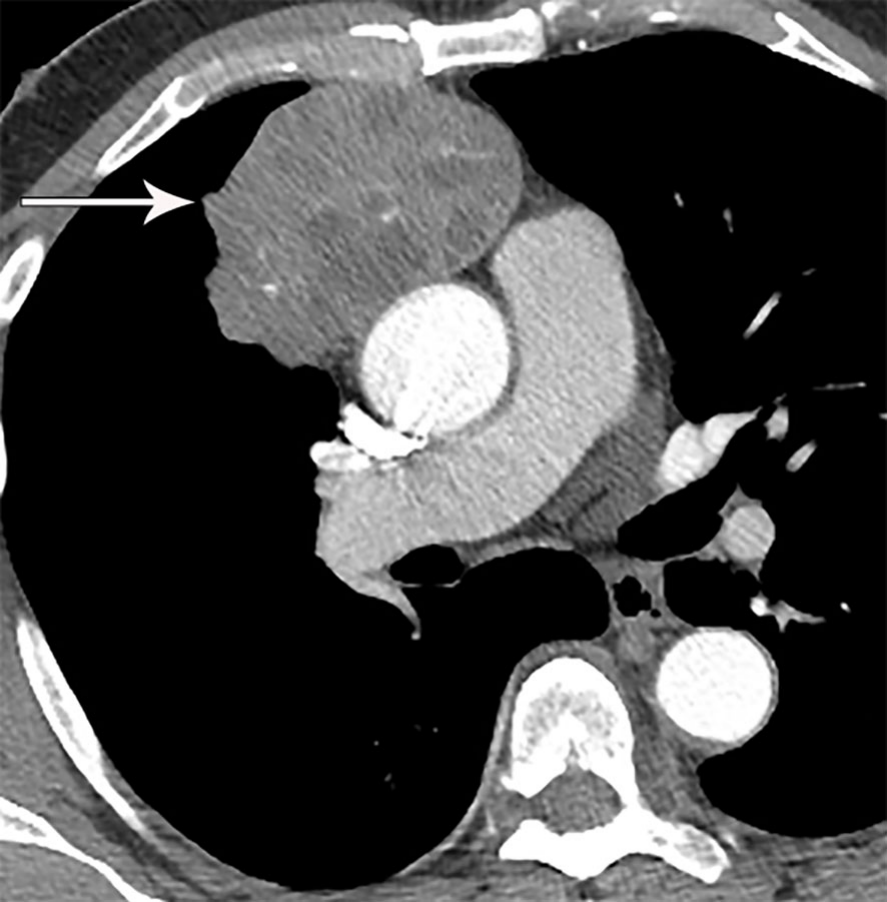
70 year old man with WHO type B3 thymoma. Contrast-enhanced CT shows right prevascular mediastinal mass with heterogeneous attenuation and areas of necrosis (arrow), consistent with more aggressive WHO subtype identified pathologically.

Thymoma typically presents on CT as a smooth or lobular mass involving one lobe of the thymus, with bilateral involvement more rarely occurring (40). The majority of thymomas demonstrate homogeneous contrast enhancement, however, approximately one third are more heterogeneous due to areas of hemorrhage, necrosis, or cystic change with punctate, linear capsular, or coarse intratumoral calcifications possible (1) (**Figures 9**, **10**). CT characteristics of thymoma can vary according to lesion grade, with vascular invasion, pleural and pericardial involvement more common with higher-grade lesions (**Figure 2**). While imaging overlap is present, higher grade tumors tend to be larger, have lobular or irregular contour, areas of cystic or necrotic change, areas of calcification, and evidence of infiltration of surrounding fat (41–43) (**Figure 11**).

On MRI, thymomas present as prevascular masses with low to intermediate signal intensity on T1 weighted images and high signal intensity on T2 weighted images with areas of cystic change or necrosis presenting as decreased T1 signal intensity and increased T2 signal intensity. Fat suppression imaging can be utilized to better delineate thymomas from surrounding mediastinal fat which facilitates more exact measurements and evaluation of enhancement. When compared to CT, MRI is more limited in the detection of areas of calcification. However, MRI excels in identifying nodules, thickened septae, and/or thickened capsule seen in cystic thymoma and differentiating this from a benign prevascular cyst. MRI also excels in identifying direct cardiac involvement due to its improved contrast resolution as compared to CT (44).

The role of PET/CT in thymoma imaging is limited by the presence of FDG uptake in the normal and hyperplastic thymus, especially in younger adults and children. Physiologic uptake in the thymus has been reported in 28% of patients under 40 years of age and up to 73% in children less than 13 years of age (45). While studies are small, PET/CT has not been demonstrated to reliably differentiate various grades of thymic tumors, although, higher grade tumors do tend towards higher FDG activity (46, 47) (**Figures 2**, **3**). Indium-^111^ octreotide nuclear medicine scans, previously used to demonstrate which tumors may respond to octreotide, which is a second or third line therapy after chemotherapy failure (48), have now been replaced by ^68^Ga-labeled somatostatin analogues. This is because these ^68^Ga-labeled somatostatin analogues, such as ^68^Ga-DOTATATE, are used to image with a PET/CT scanner providing better resolution.

## Thymic Carcinoma and Neuroendocrine Tumor/Carcinoid

**Figure 12:**
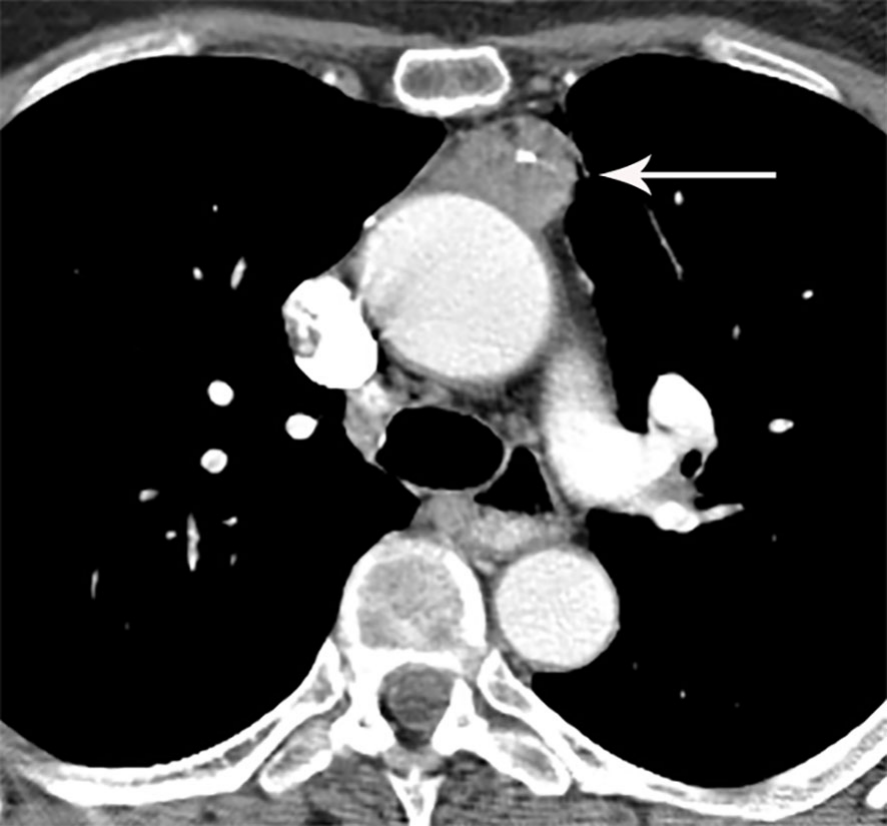
69 year old woman with thymic carcinoma. Contrast-enhanced CT shows left prevascular mediastinal mass (arrow) with small calcific focus.

**Figure 13:**
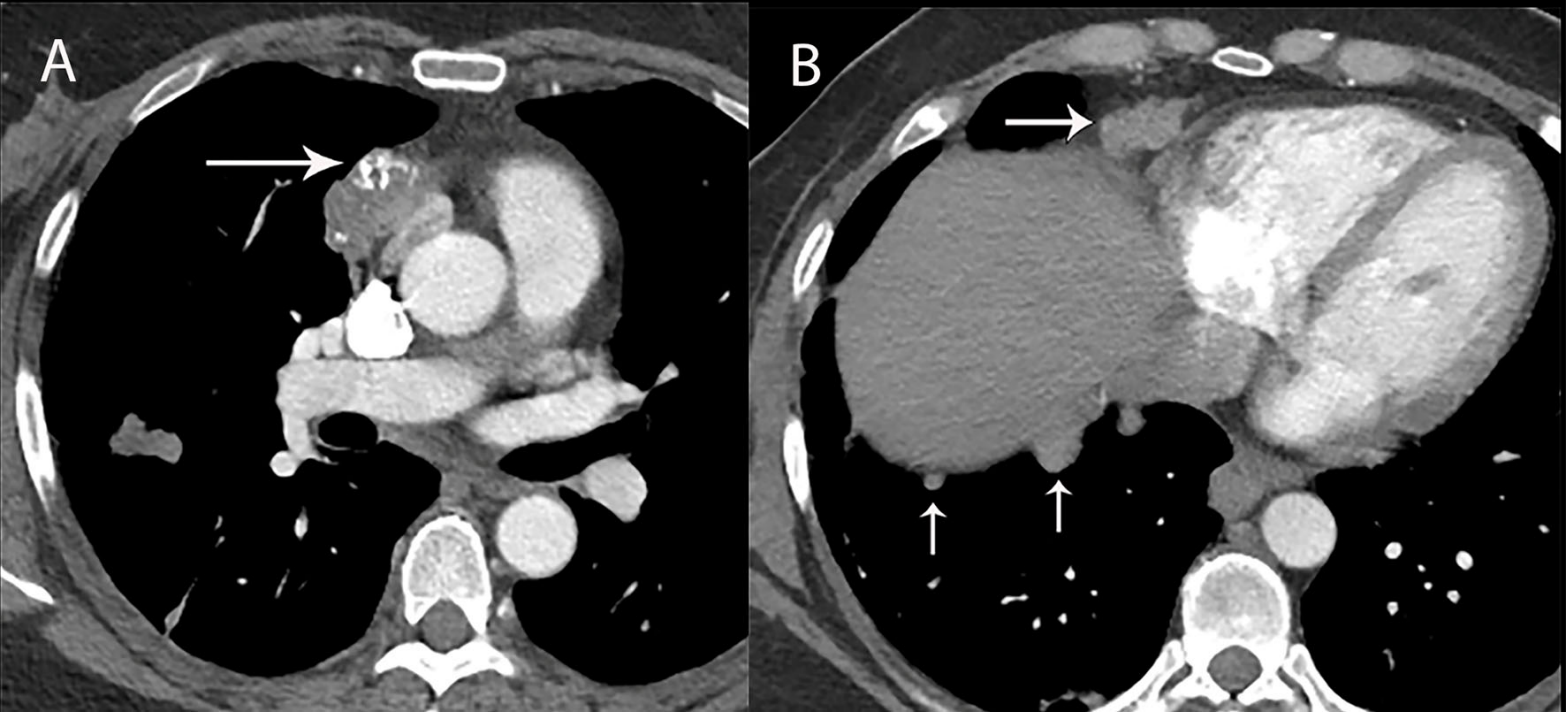
46 year old woman with thymic carcinoma with pleural metastases. **(A)** Contrast-enhanced CT shows right prevascular mediastinal mass (arrow) with heterogeneous attenuation and calcifications. **(B)** CT shows nodular right diaphragmatic pleural metastases (vertical arrows) and right anterior diaphragmatic nodal metastasis (horizontal arrow).

**Figure 14:**
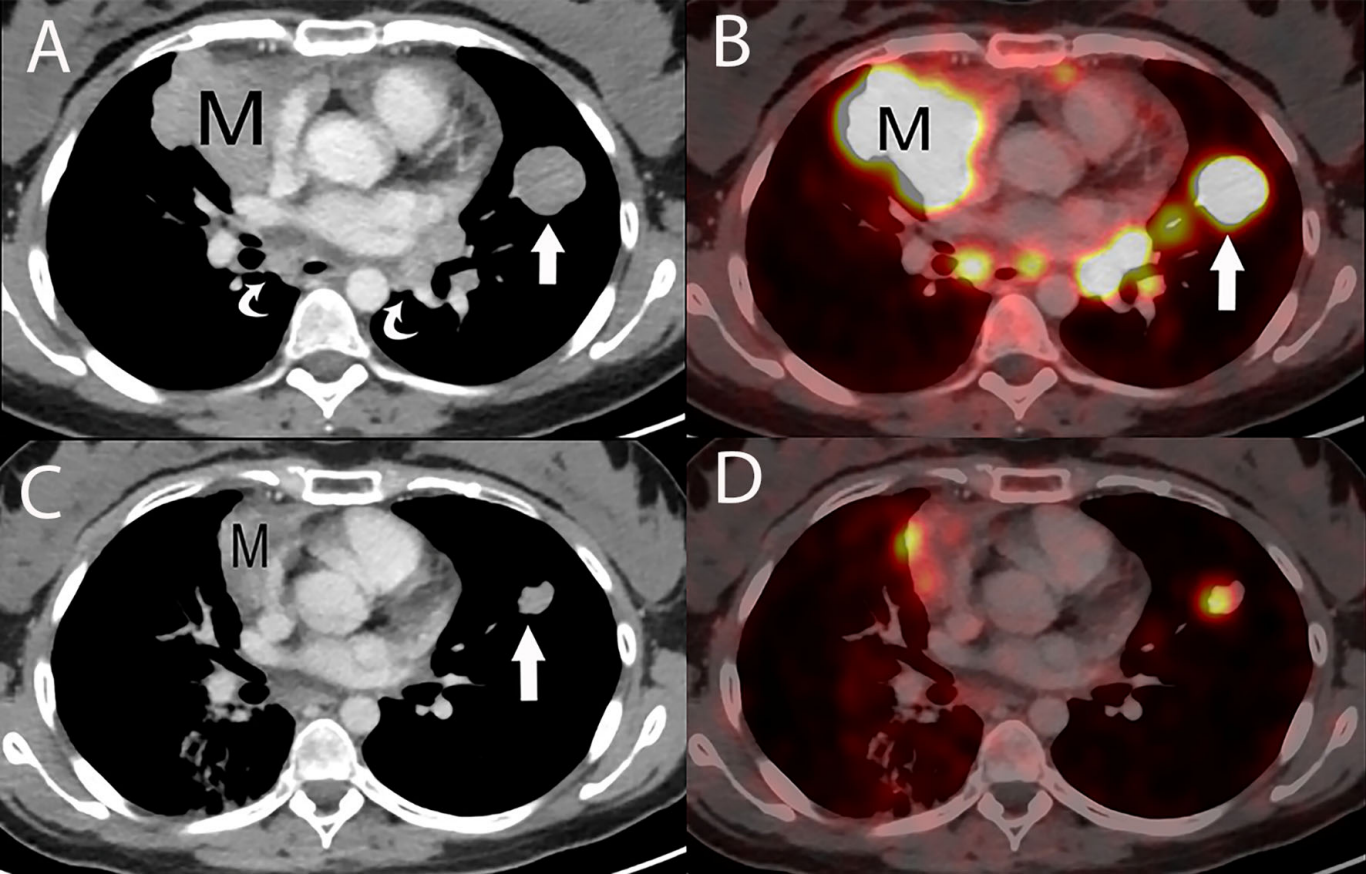
29 year old woman with metastatic thymic carcinoma treated with peptide receptor radiotherapy (PRRT) with ^177^Lutetium. **(A)** Baseline contrast enhanced chest CT prior to PRRT treatment shows the primary prevascular mass (M), lung metastasis (straight arrow) and metastatic mediastinal lymphadenopathy (curved arrows). **(B)** Axial fused PET/CT scan at the same level as A, using a somatostatin analogue, Ga ^68^-DOTATATE, reveals DOTATATE uptake in the primary mass (M), lung metastasis (straight arrow) and mediastinal lymphadenopathy. **(C)** Contrast enhanced chest CT, following two peptide receptor radiotherapy (PRRT) with ^177^Lutetium sessions show an impressive partial response with a decrease in size of the primary mass (M) and of the lung metastasis (straight arrow), with resolution of the mediastinal lymphadenopathy. **(D)** Axial fused Ga ^68^-DOTATATE PET/CT scan at the same level as C, shows a corresponding marked decrease in DOTATATE uptake consistent with an impressive metabolic partial response.

Thymic carcinoma and thymic neuroendocrine tumors have similar imaging characteristics that can overlap with higher-grade thymomas, and are thus described together. Thymic carcinomas present as large prevascular masses with irregular poorly marginated borders, demonstrate a greater degree of necrosis, cystic change, and hemorrhage, with greater local invasion when compared to thymomas (1) (**Figures 2**, **12**). Evidence of pleural or pericardial nodules, pleural effusion, or distant metastasis suggests thymic carcinoma or thymic neuroendocrine tumor as compared to thymoma (**Figures 13**, **14**).

In particular, aggressive thymic epithelial tumors can commonly invade or extrinsically compress the superior vena cava (SVC) resulting in SVC syndrome, which is a clinical syndrome marked by swelling of the neck, face, and upper extremities, cough, headache, and shortness of breath.

MRI imaging findings of thymic carcinoma and thymic neuroendocrine tumors are similar as described with thymomas. Thymic carcinomas, however, tend to have a more irregular contour, greater heterogeneity related to hemorrhage, necrosis, and cystic change, greater degree of local vascular and mediastinal invasion, and lymphadenopathy (44, 49, 50).

While FDG PET/CT does not reliably differentiate various grades of thymoma, several studies of up to 112 patients suggest that PET/CT can be utilized to differentiate thymoma from thymic carcinoma using various cutoffs of SUV max ranging between 4.6 and 6.3 (51, 52). Given increased levels of FDG uptake in higher grade tumors, FDG PET/CT can be useful in the assessment and follow-up of thymic carcinoma (53). Thymic neuroendocrine tumors can also be evaluated with ^68^Ga-DOTATATE PET/CT which may demonstrate improved sensitivity for lesion detection compared with FDG PET/CT and can additionally identify tumors that are candidates for peptide receptor radiotherapy (PRRT) with ^177^Lutetium (53) (**Figure 14**).

## Primary Thymic Salivary Gland Tumors

Primary salivary gland tumors of the thymus are quite rare and must be differentiated from metastasis by detailed radiographic and clinical evaluation (54). Only a few dozen cases of primary thymic adenoid cystic carcinoma and mucoepidermoid carcinoma have been reported (55–57). Primary thymic salivary gland tumors have similar imaging characteristics as other thymic epithelial neoplasms with final diagnosis depending on histochemical evaluation.

## Other Prevascular Mediastinal Masses

Other prevascular mediastinal masses can have overlapping imaging features with thymic epithelial tumors. Hodgkin and non-Hodgkin lymphomas can be seen in the prevascular space. In distinction from higher grade thymic epithelial tumors, however, lymphoma generally presents as a homogeneous smooth or lobulated soft tissue mass that may surround, but rarely invades, adjacent structures. Calcifications are rare in untreated lymphoma.

Additionally, a variety of germ-cell tumors including benign mature teratoma and malignant embryonal carcinoma, yolk sac tumor, choriocarcinoma, and mixed germ cell tumor can be seen in the prevascular space. Heterogeneously enhancing masses with areas of cystic change, necrosis, and calcification have overlapping imaging features with thymic epithelial tumors.

## Advanced Imaging Options

### CT

A variety of CT techniques are being evaluated to better distinguish various prevascular mediastinal masses. CT perfusion, a functional imaging technique, provides quantitative data on tissue perfusion by acquiring specific graphs for tissue blood flow (BF), blood volume (BV), and permeability surface (PS) which can be used to evaluate tumor angiogenesis, tumor infiltration, and response to therapy (58). Bakan et al. found that while CT perfusion values were not significantly different between thymoma and thymic hyperplasia, there were significant differences in BF and BV values between thymomas and malignant prevascular lesions, such as thymic carcinoma, demonstrating the role of CT perfusion imaging to aid in differentiation between thymoma and thymic carcinoma (59).

Dual-energy computed tomography (DECT) has been evaluated to differentiate prevascular masses with malignant tumors revealing higher iodine concentrations (IC) as compared to benign tumors (60). Testing this on 37 patients, Chang et al. found that iodine related Hounsfield units (IHU) and IC were different among low-risk thymomas, high-risk thymomas, and thymic carcinomas, with lower values noted in higher grade tumors, presumably due to the presence of necrosis (61). They concluded that DECT, using iodine concentration measurement derived quantitative analysis, could help differentiate between low-risk thymoma, high-risk thymoma, and thymic carcinoma.

### MRI

It has been noted that routine contrast enhanced MRI cannot reliably differentiate low-risk from high-risk thymic epithelial tumors (50). Diffusion-weighted imaging (DWI) and apparent diffusion coefficient (ADC) values, which are generated using DWI data, are non-invasive functional MRI techniques that allow for quantitative evaluation of thymic epithelial tumors. Razek et al. demonstrated in a group of 30 patients with thymic epithelial tumor, that an ADC cutoff value of 1.22 x 10^3^ mm2/sec could be used to differentiate low-risk thymoma from high-risk thymoma and thymic carcinoma with an 87% sensitivity, 85% specificity, and 86% accuracy (62). Generally, hot-spot regions of interest (ROI) are utilized for DWI/ADC measurements because of ease of use. However, given concern for errors in sampling, studies have shown that histogram analysis of ADC maps can be utilized for more accurate evaluation and continued ability to aid in differentiation between low-risk thymomas, high-risk thymomas, and thymic carcinomas (63, 64).

Dynamic contrast enhanced (DCE) MRI has also been studied in relation to prevascular mediastinal tumors. In a study comparing thymic epithelial tumors, lymphoma, and malignant germ cell tumors, Yabuuchi et al. found that only thymic epithelial tumors demonstrated a washout pattern on DCE-MRI, possibly due to high tumor cellularity and limited stroma, as compared to other tumor types (65, 66). Similarly, in a study evaluating the ability of DCE-MRI to differentiate thymic carcinoma from thymic lymphoma in 29 patients, Shen et al. reported that reflux rate constant from the extracellular extravascular space (EES) to the blood plasma (k _ep_) was lower in thymic carcinoma and that the volume fraction of the EES (v _e_) was higher in thymic carcinoma as compared to thymic lymphoma (67). While these results are interesting and promising, further research is needed to clarify if DCE-MRI can be utilized to differentiate between various grades of thymoma and between thymoma and thymic carcinoma. Finally, early work reveals that quantitative features derived from MRI images are related to biologic behavior and radiomics models (advanced mathematical analysis of existing data) could help facilitate predictions of pathologic classification and staging of thymic epithelial tumors (68).

## Conclusion

Imaging plays an integral role in the management of patients with thymoma and thymic carcinoma. Imaging is used in the initial diagnosis and staging of patients, particularly in the detection of locally invasive disease and distant metastasis, to stratify patients for therapy, and to determine prognosis. Following various modalities of therapy, imaging serves to assess treatment response and to detect recurrent disease. While imaging findings overlap, a variety of CT, MRI, and PET/CT characteristics can help differentiate thymoma and thymic carcinoma, with new CT and MRI techniques currently under evaluation showing potential.

## Author Contributions

CS wrote the first draft of the manuscript. All authors contributed to obtaining images, manuscript revision, read, and approved the submitted version.

## Conflict of Interest

EM received hononaria from Merck Sharp & Dohme and Boehringer Ingelheim for giving lectures.

The remaining authors declare that the research was conducted in the absence of any commercial or financial relationships that could be construed as a potential conflict of interest.

## Publisher’s Note

All claims expressed in this article are solely those of the authors and do not necessarily represent those of their affiliated organizations, or those of the publisher, the editors and the reviewers. Any product that may be evaluated in this article, or claim that may be made by its manufacturer, is not guaranteed or endorsed by the publisher.
